# Global MicroRNA Characterization Reveals That miR-103 Is Involved in IGF-1 Stimulated Mouse Intestinal Cell Proliferation

**DOI:** 10.1371/journal.pone.0012976

**Published:** 2010-09-23

**Authors:** Yalin Liao, Bo Lönnerdal

**Affiliations:** Department of Nutrition, University of California Davis, Davis, California, United States of America; INSERM U1016, Institut Cochin, France

## Abstract

MicroRNAs play extensive roles in cellular development. Analysis of the microRNA expression pattern during intestinal cell proliferation in early life is likely to unravel molecular mechanisms behind intestinal development and have implications for therapeutic intervention. In this study, we isolated mouse intestinal crypt cells, examined the differences in microRNA expression upon IGF-1 stimulated proliferation and identified miR-103 as a one of the key regulators. Mouse intestinal crypt cells were cultured and treated with IGF-1 for 24 h. MicroRNA microarray showed that multiple microRNAs are regulated by IGF-1, and miR-103 was the most sharply down-regulated. Expression of miR-103 in mouse intestinal crypt cells was confirmed by real-time Q-PCR. Sequence analyses showed that, among the 1040 predicted miR-103 target genes, CCNE1, CDK2, and CREB1 contain complementary sequences to the miR-103 seed region that are conserved between human and mouse. We further demonstrated that miR-103 controls the expression level of these three genes in mouse crypt cells by luciferase assay and immunoblotting assay. Taken together, our data suggest that in mouse intestinal crypt cells, miR-103 is part of the G1/S transition regulatory network, which targets CCNE1, CDK2, and CREB1 during IGF-1 stimulated proliferation.

## Introduction

The small intestinal epithelium is a major site for nutrient absorption and also serves as an important barrier to prevent exogenous pathogens from entering the body. The small intestine is also a highly dynamic and well-structured tissue which compartmentalizes into villi and the crypts of Lieberkuhn (crypts). The intestine regenerates itself throughout the life as the intestinal epithelial cells regularly shed off from the villi. This continuous cell renewal process is achieved by pluripotent epithelial stem cells which populate the specialized proliferative units known as the crypts. The crypts are localized at the intervillus region, and formed as the result of epithelial invaginations towards the basolateral side of the epithelium. Infancy is a critical period to establish the proliferative potential of the crypts, and thus the maintenance of the structural and functional homeostasis in the intestine. Rodent studies have revealed that crypt structures form during the first few days after birth, and continue to develop during the next several weeks. Recently, Cummins et al. [1] found that crypt fission (also called branching, which is a process of physiologic mechanism of crypt reproduction) is present predominantly during infancy, but not during later developmental stages, further supporting the significance of infancy for crypt development.

Growth factors are known to be present in breast milk [2], [3], [4], and have been investigated in their capacities to enhance intestinal growth. Transforming growth factor alpha, hepatocyte growth factor [5], and epidermal growth factor [6] are able to significantly stimulate crypt cell proliferation as measured by ^3^H-thymidine incorporation assay. Crypt cell migration and cell proliferation increased after mucosal injury in rat crypt IEC-6 cell in response to insulin like-growth factor 1 [7]. In addition, tyrosine phosphorylation of MAPK, MAPK-dependent increase in p21 (waf1/cip1), c-Myc, and c-Fos expression were found to be upstream events in growth factor induced crypt cell proliferation [6], [8], [9]. This intricate cellular crosstalk occurred during intestinal cell growth, and is likely to involve several signaling pathways mediated via transcription factors, extracellular matrix components, and cytokines. Understanding the molecular mechanisms regulating crypt proliferation may help the discovery of more targeted strategies to promote intestinal growth as well as defining the pathologies of a number of human gastrointestinal diseases, including infection, irritable bowel syndrome, and colorectal cancer associated with aberrant patterns in crypt cell proliferation [10], [11].

MicroRNAs (miRNAs) are a new class of small non-coding RNAs emerged over the past several years, functioning as critical regulators of gene expression. MiRNAs are 19–25 nucleotides in length, highly conserved across species, and have different complementarity with their corresponding mRNAs. MiRNAs negatively regulate gene expression post-transcriptionally by repressing translation or targeting mRNA degradation [12]. MiRNAs have been shown to play crucial roles in biological processes, including the cell cycle and apoptosis [13], [14]. Our recent data revealed that miR-30e is a downstream target of beta-catenin during intestinal crypt cell differentiation [15]; Hino et al. showed that miR-194 expression was induced by HNF-1alpha during intestinal epithelial cell differentiation [16], suggesting active roles for miRNAs during intestinal development. Despite these findings, very few mechanistic studies have been performed to examine functional roles of miRNAs in intestinal cell proliferation.

The objective of this study was to use isolated mouse small intestinal crypt cells to observe functional expression of miRNAs during the IGF-1 induced intestinal crypt cell proliferation, and to identify individual miRNAs associated signaling molecules involved in this process.

## Materials and Methods

### Mouse intestinal crypt cell culture

This study complied with the *Guide for the Use and Care of Laboratory Animals* and the protocol was approved by the Institutional Animal Care and Use Committee at University of California, Davis, which is accredited by the American Association for the Accreditation of Laboratory Animal Care (AAALAC). Virgin 5–6 weeks old C57BL/6 mice were obtained from Charles River Laboratories and fed control chow diet upon arrival. Mice were maintained in stainless-steel hanging cages on a 12∶12-h light-dark cycle. Female mice were mated overnight, and the day pups were born were considered postnatal day (PD) 0. Animals aged PD10 were killed by decapitation.

Small intestine crypt cell isolation was performed according to the method of Sato et al. [17] with modifications. Briefly, small intestine was quickly isolated and the attached fat was removed. The longitudinally opened small intestine was washed with cold 1× PBS (all PBS was DEPC treated, unless otherwise noted), then transferred to a 50 ml tube with cold PBS. The intestine was washed until the supernatant was clear. The intestine was further washed with 2 mM EDTA in PBS at 4°C for 45 min. The small intestine was resuspended with new PBS, supernatant was discarded, this step was repeated and the crypt enriched portion of fractions 2–4 was used.

The crypt fraction was diluted with Advanced DMEM/F12 (ADF) and centrifuged at 1200× *g* for 5 min. The pellets were resuspended in 15 ml ADF and collected in a 50 ml falcon tube after passing through a 70 µm cell strainer (Fisher). The tube was centrifuged at 600× *g* for 2 min to remove single cells. The ADF wash was repeated 3 times. The cell pellets were resuspended with crypt culture media (Advanced DMEM/F12, GlutaMax 1∶100, Hepes 10 mM, Penicillin/Streptomycin 1∶100, N2 supplement 1∶100, B27 supplement, retinoic acid free 1∶50, mouse recombinant EGF 50 ng/ml), mouse recombinant noggin 100 ng/ml, human recombinant R-spondin1 500 ng/ml, and N-Acetylcysteine 1 µM). The number of crypts was calculated using Trypan Blue exclusion assay.

### IGF-1 treatment

Crypt cells were seeded at 50% confluency per well in 6-well plate prior to treatment with 20 ng/ml IGF-1 (R&D Systems), and the control group was treated with 1× PBS alone. Cells were collected as follows: 24 h after treatment for BrdU Cell Proliferation ELISA (Roche) and immunoblotting analysis; total RNAs were collected every 3 h during a 24 h treatment period for miRNA analysis.

### Cell culture

IEC-6 cells were maintained as described by Liao and Lönnerdal [15]. HEK293 cells were maintained as described in [18].

miR-103 mimic or LNA inhibitor transfection was performed as previousely described [18]. For immunoblotting analysis, IEC-6 cells were seeded at 50% confluency per well in 6-well plate the day prior to transfection with 50 nM miR-103 inhibitor (Dharmacon). Cells were collected 48 h after transfection; for BrdU incorporation assay, IEC-6 cells were seeded at 50% confluency per well in 96-well plate 6 h prior to transfection with 50 nM miR-103 mimic (Dharmacon); 24 h after transfection, the cells were treated with 20 ng/ml IGF-1, and cell proliferation assay was performed 24 h after IGF-1 treatment.

### Bioinformatics analysis

The mature sequence of miR-103 (5′-AGCAGCAUUGUACAGGGCUAUGA-3′) was retrieved from the miRBase Sequence Database, release 14 (http://microrna.sanger.ac.uk/sequences/), and mRNA 3′UTRs of CCNE1, CDK2, and CREB1 from human and mouse were aligned with miR-103 sequence using the *Clustal*W program (http://www.ebi.ac.uk/Tools/clustalw/index.html).

The miR-103 gene targets were predicted from the MicroCosm Targets Version 5 (http://www.ebi.ac.uk/enright-srv/microcosm/cgi-bin/targets/v5/search.pl), and the 1040 resulted genes were loaded onto the Ingenuity Pathways Analysis software (IPA version 7.6, http://www.ingenuity.com). Core Analysis was performed to examine biological networks potentially associated with these miRNAs. Futhermore, Target Scan version 5.1 (www.targetscan.org) was used to scan for seed matches between the miR-103 seed region and the predicted gene.

### MicroRNA array

RNA integrities were examined on Bioanalyzer for quality control at the University of California, Davis Genome Center. For analyzing miRNA expression, miRNA microarray was performed at the J. David Gladstone Institute Genomics Core facility, University of California, San Francisco (http://www.gladstone.ucsf.edu/gladstone/php/?sitename=genomicscore).

300 ng of each sample was labeled with Cy5/Cy3 using miRCURY LNA microRNA array Power Labeling kit (Exiqon). Hybridization SureHyb chamber kit and Gasket slide kit (Agilent) were used to hybridize the labeled RNA for 18 h to Exiqon miRCURY LNA miRNA array V.11.0 (miRBase Sequence Database, http://microrna.sanger.ac.uk/sequences, release 11.0). This array contains 9360 probes, 3300 of which represent 825 human miRNAs with 4 duplicate probes per miRNA, additional 1400 probes for miRNA in mouse or rat and 472 probes for miRNA in human/mouse viruses. In addition, there is a variety of control and other probes: 1640 empty, 476 spike_control, 28 negative controls, 12 U6-snRNA, 60 hsa_SNORD, 1728 miRPlus, 4 5SrRNA, and 240 Hy3. For each group, 3 arrays were run, and 1 array was dye swapped. Arrays were scanned on an Axon GenePix 4000B scanner, and GPR files containing fluorescent ratios (sample/control) were generated using GenePix Pro 6.0 software.

### Vector construction

A 532 bp fragment of the human CCNE1 mRNA 3′UTR (corresponding to nt 1406–1937 of the Entrez PubMed transcript BC035498) was PCR amplified from a pOTB7 vector containing CCNE1 full length cDNA (Open Biosystems); a 1426 bp fragment of the human CREB1 mRNA 3′UTR (corresponding to nt 1180–2606 of the Entrez PubMed transcript BC095407) was PCR amplified from a pBluescriptR vector containing CREB1 full length cDNA (Open Biosystems); The PCR products were cloned into pGL3-control luciferase reporter vector (Promega) via an *Xba*I restriction site, immediately downstream of the luciferase gene; a 38 bp fragment of the human CDK2 mRNA 3′UTR (corresponding to nt 1531–1568 of the Ensembl transcript BC093646) was synthesized (Genscript Inc. USA), and the gene was then directly annealed to pGL3-control luciferase reporter vector at the *Xba*I restriction site.

A 530 bp fragment of the mouse CCNE1 mRNA 3′UTR (corresponding to nt 1426–1955 of the Entrez PubMed transcript NM_007633.2) was PCR amplified from a pCMV-SPORT6.1 vector containing mouse CCNE1 full length cDNA (imaGenes); a 1160 bp fragment of mouse CDK2 mRNA 3′UTR (corresponding to nt 1227–2386 of the Entrez PubMed transcript BC005654) was PCR amplified from pCMV-SPORT6 vector containing mouse CDK2 full length cDNA (Open Biosystems); a 899 bp fragment of mouse CREB1 mRNA 3′UTR (corresponding to nt 1143–2041 of the Entrez PubMed transcript BC021649) was PCR amplified from pCMV-SPORT6 vector containing mouse CREB1 full length cDNA (Open Biosystems). The PCR products were cloned into pGL3-control luciferase reporter vector (Promega) via an *Xba*I restriction site, immediately downstream of the luciferase gene.

### Luciferase assay

For luciferase assays, HEK293 cells were seeded at 70% confluency per well in 96-well plate 6 h prior to transfection with 4 ng/µl luciferase expression construct and 12.5–80 nM miR-103 mimic (Dharmacon). Mock transfected cells were transfected with luciferase expression construct alone. cel-miR-67 (Dharmacon) served as a negative control for miR-103 mimic. Luciferase activity was measured 24 h after transfection using the Dual Glo Luciferase Assay System (Promega), and pGL4.73[hRluc/SV40] vector (Promega) served as internal control.

### Real-time Q-PCR

Total RNA from cultured mouse small intestinal crypt cells was isolated using miRNeasy Mini Kit (Qiangen) and diluted to 2 µg/µl in DEPC-treated nuclease free water (Ambion Inc.).

For miR-103 assay, cDNA was generated from 2 µg RNA using TaqMan® MicroRNA Reverse Transcription Kit (Applied Biosystems) according to the manufacturer's protocol. The mature miR-103 gene specific stem-loop RT primer for reverse transcription was designed according to miRNAs sequences listed in the Sanger miRBase (http://microrna.sanger.ac.uk/sequences/). The pri-miR-103 assay kit was purchased from Applied Biosystems. The primers used for β-actin were forward 5′-ACTGCTCTGGCTCCTAGCAC-3′, reverse 5′-ACATCTGCTGGAAGGTGGAC-3′. The RT reaction and PCR reaction were performed as previously described [18].

Each sample was analyzed in triplicate and normalized to snoRNA202 for mature miR-103 and β-actin for pri-miR-103 using the following equation: ΔCt_GENE_ = Ct_GENE_-Ct_snoRNA202/β-actin_. The fold change, relative to the control group was calculated using the following equation: 2^(ΔΔCtGENE)^ where ΔΔCtGENE = ΔCt_GENE_ of snoRNA202/β-actin - ΔCt_GENE_ of each well.

### Immunoblotting analysis

Mouse intestinal crypt cells before and after IGF1 treatment were homogenized in RIPA buffer (25 mM Tris-HCl pH 7.6, 150 mM NaCl, 1% NP-40, 1% Sodium deoxycholate and 0.1% SDS) containing 1× complete EDTA-free protease inhibitor (Roche). 50 µg proteins were electrophoresed through 10% polyacrylamide gel, transferred onto nitrocellulose membrane at 350 mA for 60 min, and blocked overnight in 1× PBS/0.1% Tween-20 (PBST) with 5% non-fat milk at 4°C.

Bands were detected using Super Signal Femto chemiluminescent reagent (Pierce) and quantified using the Chemi-doc gel quantification system (Bio-Rad). All data were normalized to mouse β-actin.

### Data analysis

GPR files were read into R/Bioconductor [19] using the marray package. GenePix flagged spots were removed from subsequent analysis, and only unflagged human, mouse and rat probes were used for normalization and subsequent analysis. M (log2 ratios) of Cy5 to Cy3 signals were calculated for each array, and normalized by print tip loess normalization [20], [21] using only unflagged spots. For each miRNA with more than 1 unflagged probe, the median of normalized M of the replicate probes was taken as its summary value for the miRNA in each array. The summarized M of the samples/array of each experiment was then used in moderated t-statistics and p-value calculation using the limma package in R/Bioconductor [22], [23] with adjustment for false discovery rate using the Benjamini-Hochberg method [24].

All other data were analyzed by Prism (Prism GraphPad Software). The difference between treatment group and control were tested by two-tailed Student's *t* test. Data are shown as means ± SEM of three independent experiments. Differences were considered significant when *P*<0.05.

## Results

### IGF-1 stimulates proliferation of mouse small intestinal crypt cells

**Figure 1:**
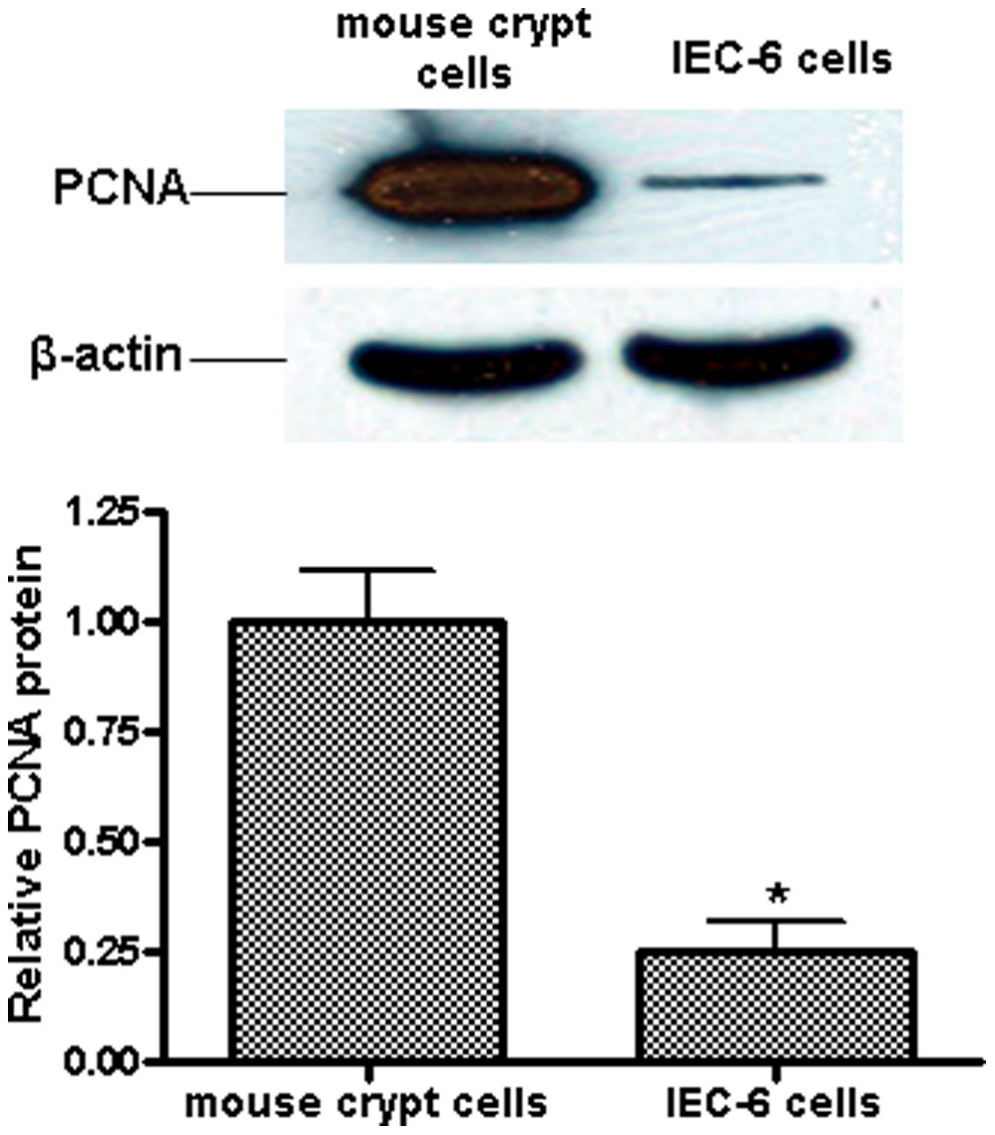
Mouse small intestinal crypt cells express PCNA. PCNA expression was analyzed by immunoblotting. 50 µg total proteins from extracted mouse small intestinal crypt cells and cultured rat intestinal crypt cell line IEC-6 were loaded. PCNA was expressed in both cell types and with significantly higher abundance in mouse small intestinal crypt cells (*P*<0.01) than in IEC-6 cells. β-actin served as a loading control. Values are means ± SEM run in triplicates.

**Figure 2:**
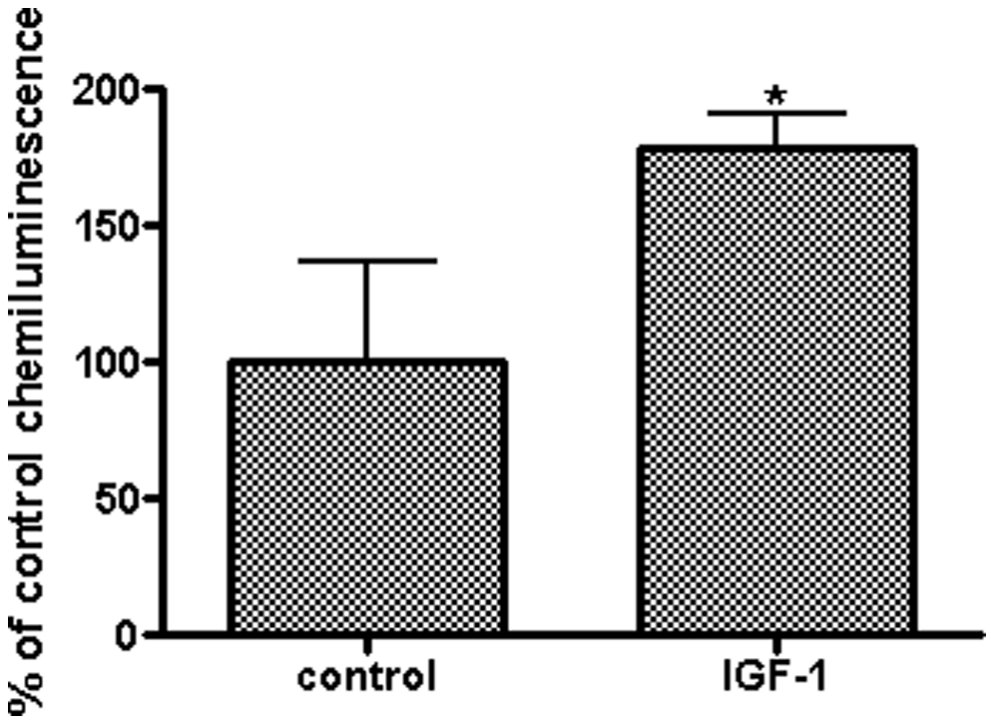
IGF-1 stimulates mouse small intestinal crypt cell proliferation. Crypt cells in 96-well plate were treated with 20 ng/ml IGF-1; the control group was grown in the crypt cell growth medium alone. After treatment for 24 h, rate of cell proliferation was measured by Brdu incorporation assay. IGF-1 significantly stimulated proliferation of the crypt cells (*P*<0.05). Values are means ± SEM run in triplicates; asterisks indicate significant differences between IGF-1 treatment and control groups.

By adapting the crypt culture protocol from Sato et al. [17], we were able to isolate mouse small intestinal crypt cells. The cells attach to the culture dish about 3 h after seeding. PCNA is highly expressed in the isolated crypt cells (Figure 1), and rat crypt IEC-6 cells served as a reference for PCNA expression. During 24 h of treatment, IGF-1 stimulated crypt cell proliferation rate (100.0%±37.1% vs 178.9%±12.5%, *P*<0.05) by the BrdU incorporation assay (Figure 2).

### Identification of microRNAs differentially regulated during IGF-1 stimulated crypt cell proliferation

**Figure 3:**
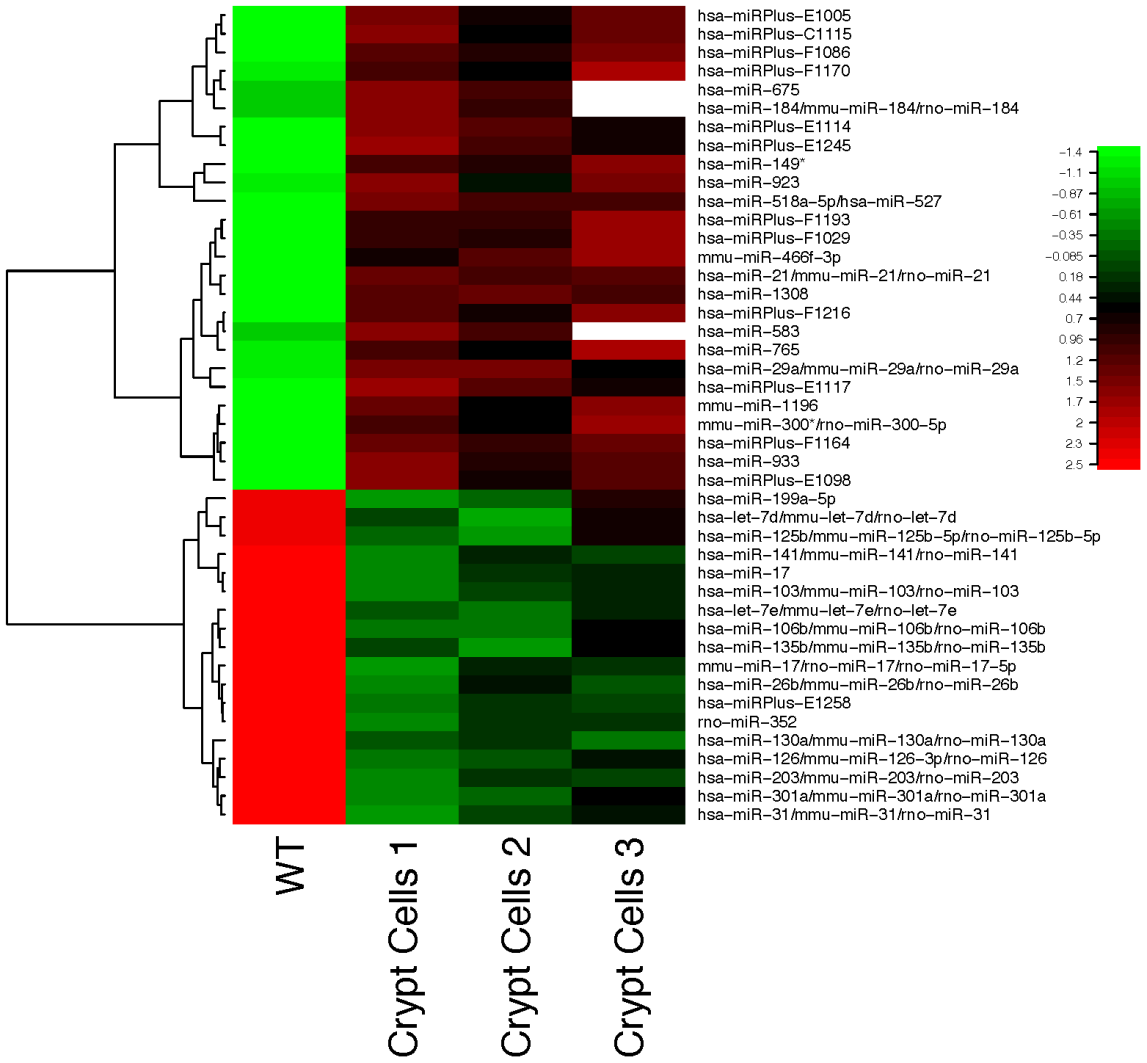
Forty-four miRNAs were significantly correlated with mouse crypt cell proliferation upon IGF-1 treatment. Hierarchical clustering analysis was performed using Euclidian distance. Each row represents relative levels of expression for a significantly regulated single microRNA and each column represents the relative expression level of a single replicate relative to control (*P*<0.01). Colors on the figure represent the scaled fold-change between samples within a gene. Control group was set to 0.

We generated a microarray containing 1293 nonredundant human, rodent, and virus miRNA species to determine expression levels of mature miRNAs purified from mouse small intestinal crypt cells before and after 20 ng/ml IGF-1 treatment. The raw data are deposited in Gene Expression Omnibus (GEO), with accession number GSE20133. Among all screened individual miRNAs, 44 miRNAs were significantly regulated, including 18 significantly down-regulated, and 26 significantly up-regulated upon IGF-1 treatment (Figure 3).

### miR-103 is directly involved in IGF-1 stimulated mouse intestinal cell proliferation

**Figure 4:**
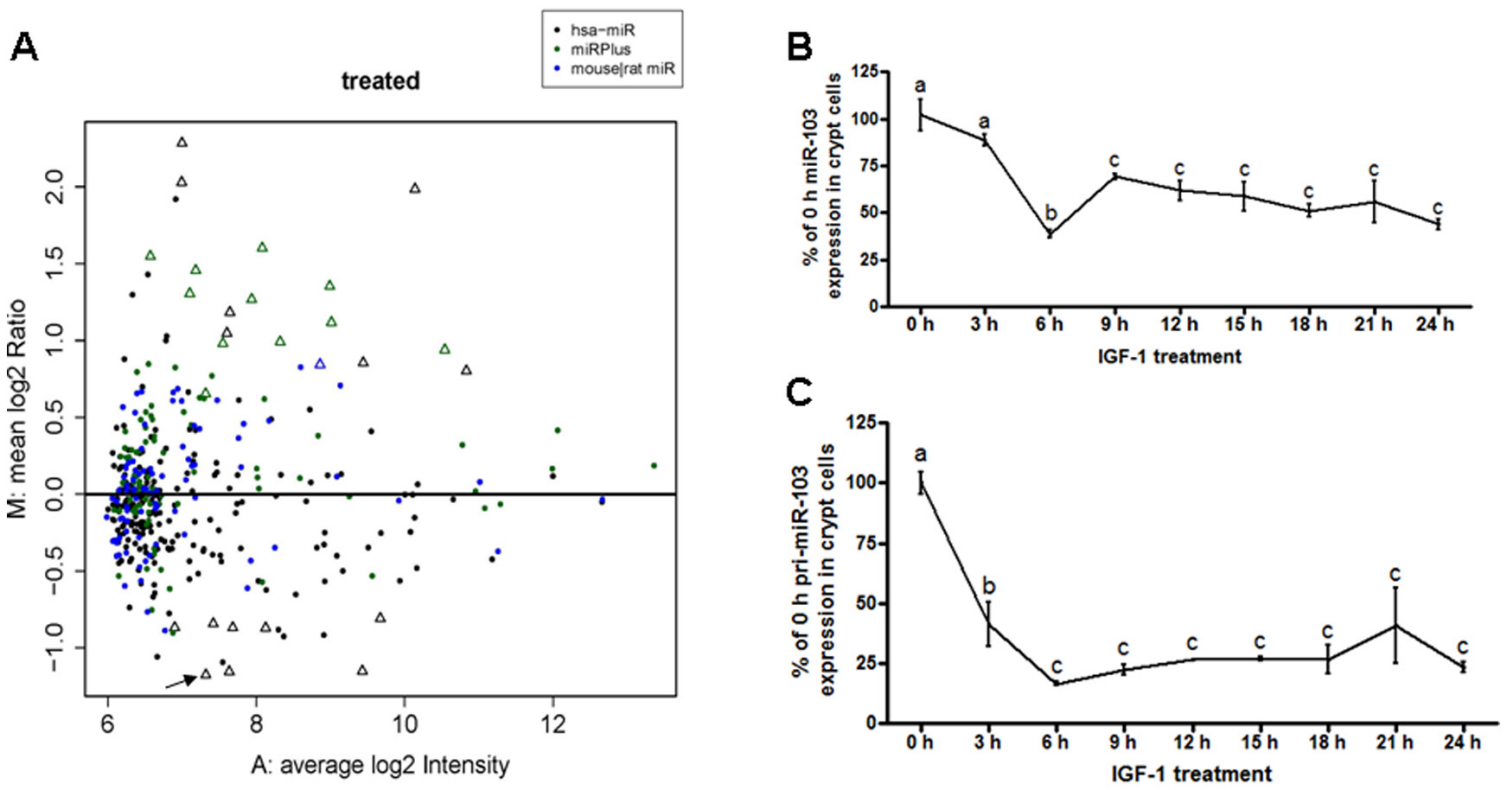
miR-103 expression analysis in IGF-1 stimulated mouse small intestinal crypt cells. (A) microrarray analysis of total miRNA expression in crypt cells after stimulation with IGF-1 for 24 h. The MA plot shows the averaged and background-subtracted fold change on a log2 scale (Y axis) and average expression intensity (X axis) of each miRNA on both channels for Cy-3 labeled control and Cy-5 labeled treated cells and their dye-swaps. Each dot or triangle represents one miRNA probe. Arrow indicates miR-103 probe, which is 44.26% of relative control intensity over the course of 24 h. (B, C) real-time Q-PCR analysis of mature miR-103 (B) and pri-miR-103 expression (C) during 24 h IGF-1 treatment in crypt cells. Data were normalized to snoRNA-202 levels for mature miR-103 and β-actin for pri-miR-103. Values are means ± SEM run in triplicates, letters indicate significant differences between time points.

**Figure 5:**
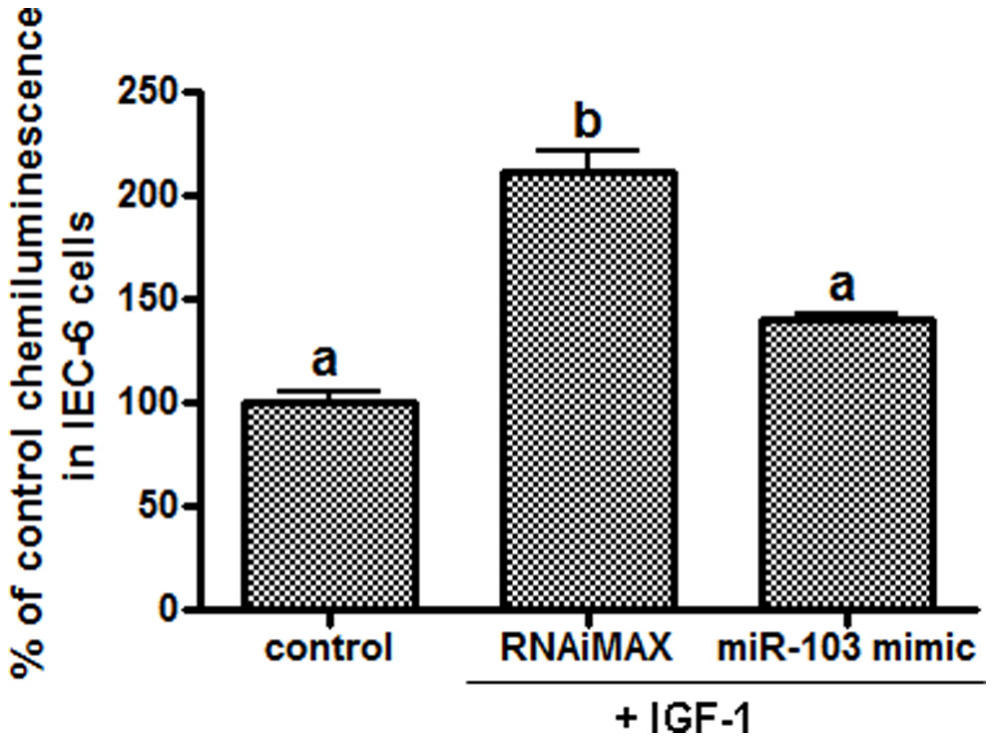
miR-103 is directly involved in IGF-1 stimulated crypt cell proliferation. IEC-6 cells in 96-well plate were transfected with 50 nM miR-103 mimic before treated with 20 ng/ml IGF-1. The control group was cultured in growth media or treated with IGF-1 alone. Cell proliferation was measured by BrdU incorporation assay. Values are means ± SEM run in triplicates. Letters indicate significant differences between IGF-1 treatment and control groups.

MA plot showed that miR-103 was the most substantially reduced miRNA during IGF-1 stimulated crypt cell proliferation (Mean log2 ratio of −1.176) (Figure 4A). Kinetic analysis by real-time Q-PCR confirmed the presence of mature miR-103 (Figure 4B) and pri-miR-103 (Figure 4C) in small intestinal crypt cells, and also revealed that during the first 6 h of IGF-1 treatment, expression of mature miR-103 and pri-miR-103 was rapidly reduced to 39% and 16% of initial levels, respectively (*P*<0.01). Mature miR-103 then rebounded to 57% of the level prior to treatment (*P*<0.01), while pri-miR-103 expression was maintained at ∼25% of the level prior to treatment (*P*<0.01). Furthermore, IGF-1 stimulated cell proliferation in IEC-6 cells was significantly inhibited by overexpression of miR-103 (211%±17% vs 140%±5%, *P*<0.05) (Figure 5).

### Identification of miR-103 target genes in mouse small intestinal crypt cells

**Figure 6:**
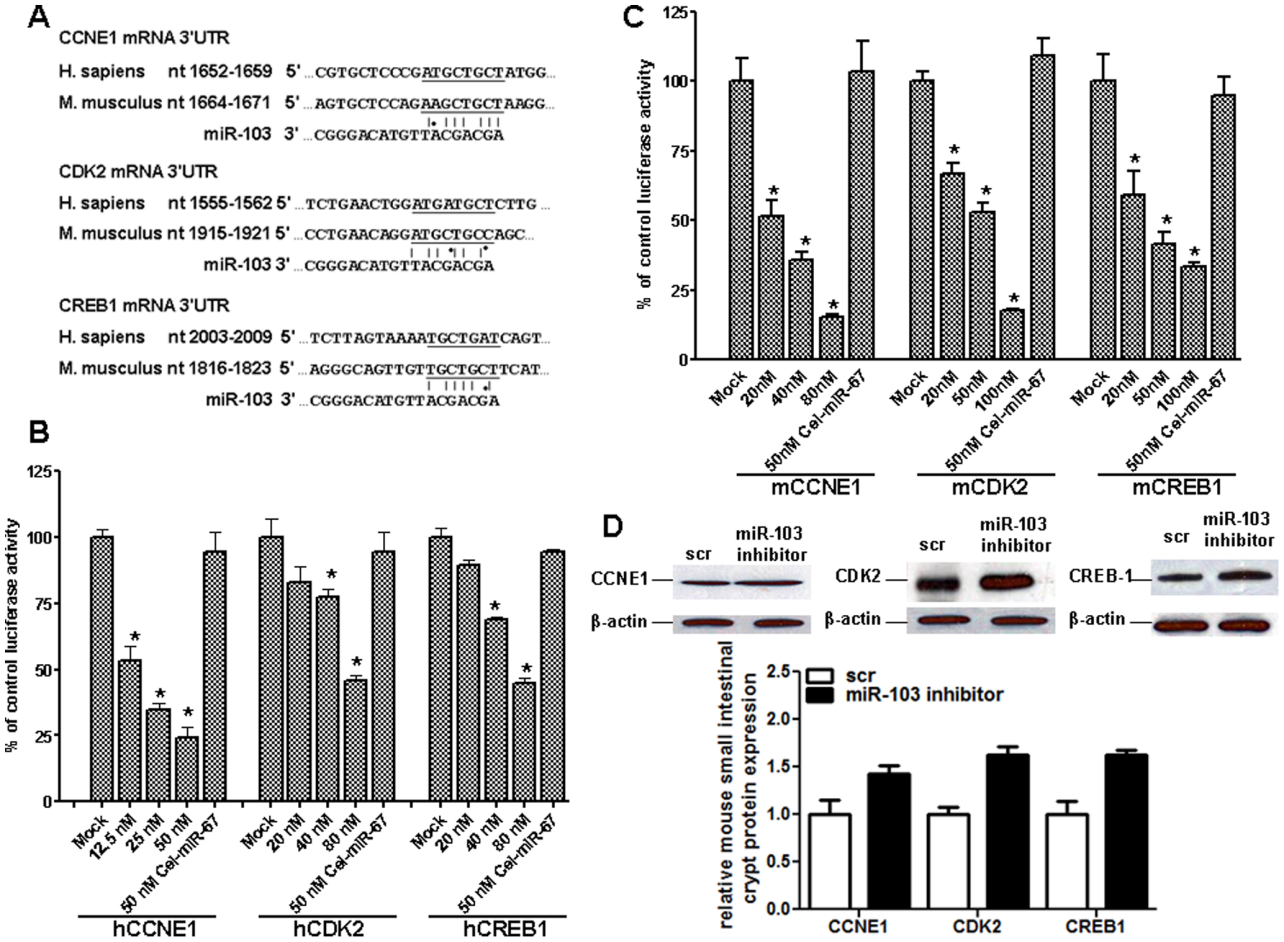
CCNE1, CDK2, and CREB1 are direct targets of miR-103 in mouse small intestinal crypt cells during IGF-1 stimulation. (A) sequence alignment between miR-103 seed region and the seed matches on CCNE1, CDK2, and CREB1 mRNA 3′UTR region. The analyses were performed using the miRBase target database. The line indicates conserved seed match (A–T, C–G) in human and mouse, whereas the dot indicates seed match in one of the two species. (B, C) miR-103 directly binds and represses CCNE1, CDK2, and CREB1 mRNA through 3′UTR. pGL3-control luciferase vectors containing the mRNA 3′UTR of respective genes of human (B) or mouse (C) origin were co-transfected with the indicated amount of miR-103 mimic in HEK293 cells, and luciferase activity was analyzed 24 h post-transfection. The structurally unrelated miRNA cel-miR-67 served as negtive control. (D) CCNE1, CDK2, and CREB1 protein expression were examined in IEC-6 cells after transfection with 50 nM miR-103 inhibitor. Scramble (scr) miRNA transfected cells served as control. Values are means ± SEM run in triplicates, and letters indicate significant differences between treatment groups and control.

We retrieved the predicted 1040 target genes of miR-103 from the miRBase target database, and loaded them onto the Ingenuity Pathway Analysis software (IPA version 7.6). We were then able to identify cellular pathways affected by IGF-1 treatment of crypt cells. Interestingly, among the 5 top ranked molecular networks, “cell cycle, cell death” had the highest score; Remarkably, among the 5 top ranked biofunctions, “cellular growth and proliferation” involved 147 molecules, 1/3 of the total number of molecules. CCNE1 as a predicted target was the top ranked molecule with the lowest exponential value of 8.49E-09. We then used Target Scan to screen the target genes, focusing on the cell cycle and cell death regulation. Importantly, we identified CCNE1, CDK2, and CREB1 as critical components during the cell cycle process. We then manually aligned the mRNA 3′UTR and the miR-103 seed region (Figure 6A), which were found to be highly matched for both human and mouse genes.

We explored whether miR-103 could affect the identified gene targets through interaction with the mRNA 3′UTR. In HEK293 cells, co-transfection with miR-103 mimic could repress the luciferase activity generated by luciferase vectors containing the mRNA 3′UTRs of CCNE1, CDK2, CREB1 of human (Figure 6B) and mouse (Figure 6C) origin in a dose dependent manner, clearly indicating direct binding between the miRNA sequence and the genes. In comparison, the negative control miRNA, cel-miR-67, did not result in any significant reduction of luciferase activity. As expected, in IEC-6 cells, miR-103 knock down by a LNA oligonucleotide resulted in marked up-regulation of the aformentioned gene products (Figure 6D) (1.43±0.13 vs 1.00±0.26 for CCNE1, *P*<0.05; 1.63±0.13 vs 1.00±0.13 for CDK2, *P*<0.01; 1.62±0.10 vs 1.00±0.25 for CREB1, *P*<0.05), presumably because of a decrease in miR-103 mediated mRNA inhibition or degradation.

### CCNE1, CDK2, and CREB1 are directly involved in mouse intestinal cell proliferation stimulated by IGF-1

**Figure 7:**
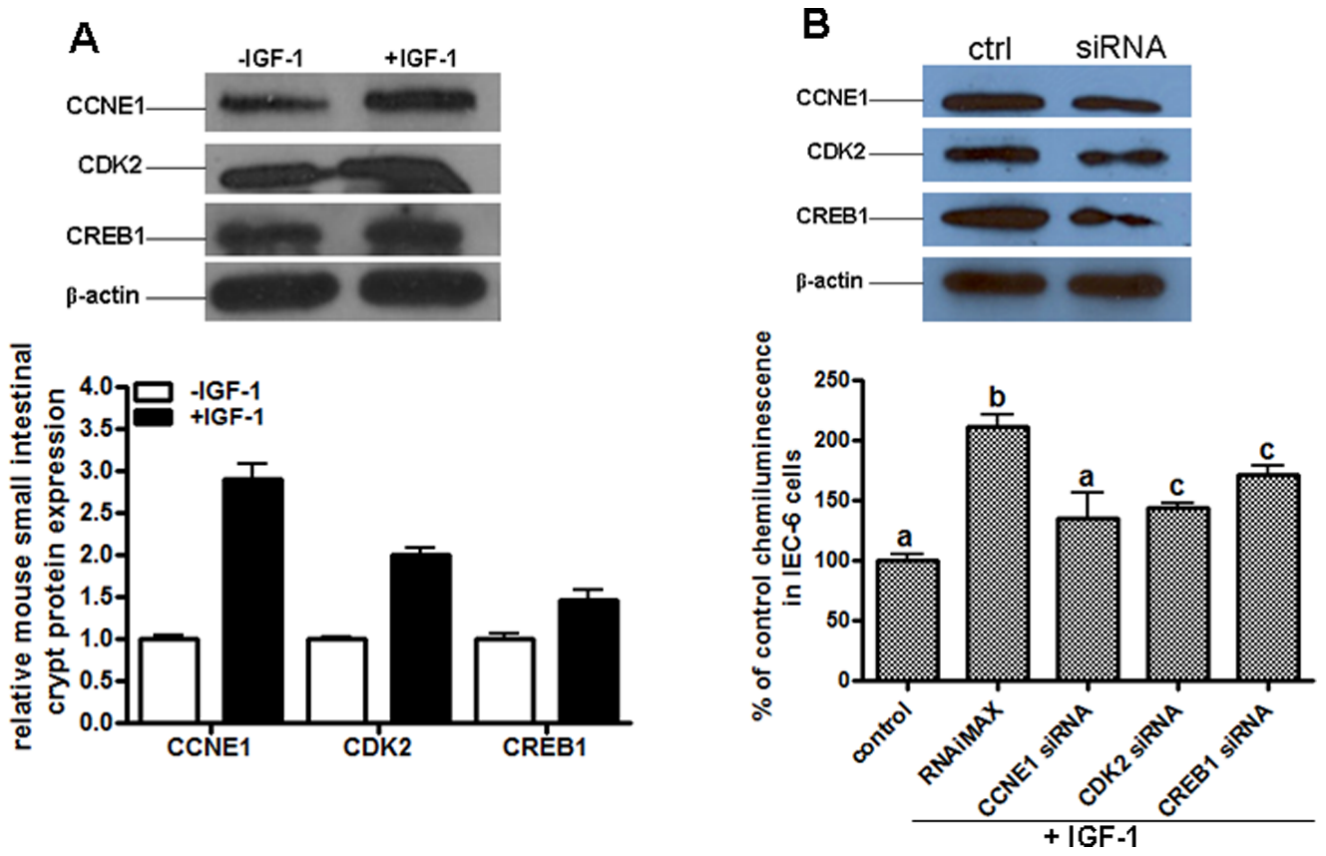
CCNE1, CDK2, and CREB1 are directly involved in IGF-1 stimulated crypt cell proliferation. (A) immunoblotting analyses of CCNE1, CDK2, and CREB1 protein in mouse small intestinal crypt cells with or without IGF-1 treatment for 24 h. Data were normalized to β-actin. Values are means ± SEM run in triplicates, asterisks indicate significant differences between control and IGF-1 treated cells. (B) CCNE1, CDK2, and CREB1 proteins were knocked-down by siRNA in IEC-6 cells before treated with IGF-1. Cell proliferation was measured 24 h after IGF-1 treatment. Values are means ± SEM run in triplicates, letters indicate significant differences between time points.

To examine whether the miR-103 targets are involved in IGF-1 stimulated mouse intestinal cell proliferation, immunoblotting analyses were performed on the crypt cells before and after IGF-1 treatment. As shown in Figure 7A, IGF-1 significantly upregulated CCNE1, CDK2, and CREB1 protein expression. In addition, IGF-1 stimulated cell proliferation of IEC-6 cells was significantly inhibited by siRNA of these genes (Figure 7B).

## Discussion

IGF-1 is a 7.5 kD polypeptide consisting of 70 amino acids, and found to be present in human milk [25]. In the gastrointestinal tract, the IGF-1 signaling pathway is initiated by the interaction between cell surface IGF-1 receptors, which are most abundant at the basolateral side of the crypt. IGF-1 is a potent stimulator of intestinal crypt cellular growth, and has been used as a therapeutic approach to facilitate the intestinal repair process in gastrointestinal disorders, such as enteritis and IBD [26]. However, few investigations have examined the molecules and signaling pathways involved in the crypt growth under normal conditions. MiRNAs are a class of small regulatory molecules which critically affect mRNA translation in a sequence specific manner. It has been shown that miRNAs control the expression of genes related to cellular development; however, to date miRNA expression in small intestinal proliferative unit crypts has not been studied and its role in crypt proliferation remains to be determined. In this study, we successfully cultured mouse intestinal crypt cells during a 24 h period and confirmed targeting of the CREB1 transcription factor, and CCNE1/CDK2 by miR-103 during cell cycle progression, implicating a role for miRNAs in intestinal growth.

There are some limited reports on intestinal epithelial miRNA expression. Takada et al. [27] showed high abundance of miR-143 and miR-194 in mouse small intestine and Hino et al. [16], [28] further showed induction of miR-194 by HNF-1 during differentiation of intestinal epithelial Caco-2 cells. We used a sensitive model for stimulating intestinal crypt cell proliferation by a growth factor, and obtained not only the first extensive mouse intestinal crypt cell miRNA profiles, but also the differentially expressed miRNA species during cell proliferation. Our screening identified a total of 233 microRNAs from mouse small intestinal crypt cells, and showed that crypt cells have dramatically different miRNA profiles upon IGF-1 stimulation. We performed real-time Q-PCR analysis to monitor the expression of some of these intestinal miRNAs, from which we confirmed two down-regulated miRNAs, miR-103 and miR-17. No statistically significant regulations were found for miR-143 or miR-194.

The miR-103 family is found in 23 species, and comprised of 2 isoforms: miR-103-1 located on human chromosome 5q34 and miR-103-2 located on human chromosome 20p13 [29], [30], [31]. Interestingly, the long arm of chromosome 5 (5q) is a region associated with the risk of developing the gastrointestinal disorders of Crohn's disease and IBS (Genetics Home Reference, http://ghr.nlm.nih.gov/). miR-103 has also been shown to stimulate adipogenesis that accelerates fat cell proliferation, and is down-regulated in obesity [32].

Remarkably, among predicted gene targets for miR-17 were WEE1, CCNA1, E2F5, MCM4, RAD51, and CABLES1, which are critically involved in cell cycle progression. For example, WEE1 is a key cell cycle regulator by the inhibiting Cdc2 at the G2/M transition, coordinating cell cycle and cell sizes; a decrease in WEE1 leads to smaller daughter cells and an increase in WEE1 prevents cells from entering mitosis [33]. CCNA1 belongs to the cyclin family, and binds to important cell cycle regulators such as E2F, Rb, CDK2 and p21, but its abundance in the intestine is low [34]. Ingenuity Pathway Core Analysis of miR-17 showed that cell cycle, DNA replication, recombination, repair, and cancer scored 34, being the second highest identified network. The G2/M transition of the cell cycle had a P-value of 7.81-E02, being one of the most significantly affected cellular processes.

In this report, we examined whether miR-103 can bind and affect the predicted target mRNAs through mRNA 3′UTR interactions. Accordingly the full-length mRNA 3′UTRs of CCNE1, CDK2 and CREB1 were individually cloned into a reporter vector, downstream of a firefly luciferase cDNA. The human homologue of the mRNA 3′UTR was used, and computational approaches (Figure 6A) predicted that there is an 8-mer seed match between miR-103 and mouse CREB1/CDK2, while there is a 7-mer seed match between miR-103 and the human homologue; there is a 7-mer seed match between miR-103 and mouse CCNE1 mRNA 3′UTR, and an 8-mer seed match between miR-103 and the human homologue. As seen in Figures 6B and Figure 6C, in HEK293 cells, all vectors containing the 3′UTRs of CCNE1, CDK2, and CREB1 displayed dose-dependent light emission reduction upon co-transfection with increasing miR-103 mimic, indicating the presence of a direct binding site for miR-103. The CCNE1 vector displayed a more sensitive response to the miR-103 mimic, possibly because of higher percentage seed match to the human homologue. Immunoblotting of IEC-6 cells demonstrated that CCNE1, CDK2, CREB1 are induced at the protein level upon miR-103 inhibition, further supporting an effect of miR-103 regulation on these genes.

CCNE1 is a member of the cyclin family which is characterized by a dramatic periodicity in protein expression throughout the cell cycle [35]. CDK2, CDK4, and CDK6 are the active kinases controlling G1/S transition in mammals. CCNE1 forms a complex with CDK2 and functions as a serine/threonine kinase regulatory subunit, which is indispensible for cell cycle G1/S progression, peaking at the G1-S phase boundary. [36], [37]. Direct functional evidence for CCNE1 is provided by the microinjection of anti-cyclin E antibodies into fibroblasts during G1 which resulted in cell cycle arrest [38]. It should be noted that other microRNAs potentially regulating CCNE1 protein expression were also significantly changed, including down-regulation of miR-141, miR-16, miR-15a, miR-352, miR-15b and up-regulation of miR-518e, miR-29a, miR-192, and miR-29b, implicating a regulatory network fine-tuning the cell cycle checkpoints.

Among the mRNA targets of miR-103 was also the transcription factor CREB1 gene, which binds as a homodimer to cAMP responsive elements within the DNA sequence [39]. Activation of CREB1 induces the early response genes, and late response genes during the early G1 phase of cell cycle [40], [41]. Remarkably, upon IGF-1 treatment, CREB1 also showed up-regulation in the TranSignal Cell Growth Protein DNA Array (Panomics Inc.), which includes AP1, c-Myb, CREB (miRBase predicted miR-103 target), E2F-1, EGR, Ets, FKHR, Myc-Max, NE-E1 (YY1), NF-κB(miRBase predicted miR-103 target), OCT-1, p53, Pax-2, Pax-3, Pax-6, PPAR-γ, Smad SBE, Sp1, SRE, and Stat3. In immunoblotting analysis, the phosphorylated-CREB1 protein was also up-regulated, indicating functional activation during miR-103 inhibition.

An improved understanding of miRNA signatures during intestinal epithelial cell proliferation should ultimately lead to the discovery of drugs better suited to treat diseases due to abberant cellular growth, such as inflammatory diseases and cancer. The present study determined the global microRNA expression in mouse crypt cells, and confirmed functional aspects of miR-103, which could be a suitable candidate for developing novel therapeutic interventions.
